# Enhancing canola oil's shelf life with nano‐encapsulated *Mentha aquatica* extract for optimal antioxidant performance

**DOI:** 10.1002/fsn3.3717

**Published:** 2023-10-06

**Authors:** Javad Tavakoli, Habib Abbasi, Sara Gashtasebi, Mohsen Salmanpour, Amin Mousavi Khaneghah

**Affiliations:** ^1^ Department of Food Science and Technology, Faculty of Agriculture Jahrom University Jahrom Iran; ^2^ Department of Nutrition Sciences, Ewaz School of Health Larestan University of Medical Sciences Larestan Iran; ^3^ Department of Chemical Engineering Jundi‐Shapur University of Technology Dezful Iran; ^4^ Department of Food Science, Engineering and Technology University of Tehran Karaj Iran; ^5^ Cellular and Molecular Biology Research Center Larestan University of Medical Sciences Larestan Iran; ^6^ Department of Fruit and Vegetable Product Technology Prof. Wacław Dąbrowski Institute of Agricultural and Food Biotechnology – State Research Institute Warsaw Poland

**Keywords:** antioxidant effect, chitosan, *Lepidium perfoliatum* gum, nano‐encapsulation, oxidative stability

## Abstract

Incorporation of antioxidants, such as phenolic compounds into edible oils has limitations such as rapid release of phenolic compounds, low solubility, low penetration, low accessibility, and rapid degradation by environmental compounds. To solve this problem, the nano‐encapsulation process is offering promising opportunities. In this research, for the first time, the phenolic extract of *Mentha aquatica* was nano‐encapsulated in nano‐emulsions coated with chitosan, *Lepidium perfoliatum* gum (LPG), and complex of chitosan and LPG (CCL) (1:1 ratio). Based on various tests (particle size measurement, ζ‐potential, polydispersity index, encapsulation efficiency index, and intensity curve), the LPG coating was the most optimum option for nano‐encapsulation compared to the other coatings. Thus, the LPG‐assisted nano‐encapsulated phenolic extract of *M. aquatica* was used to improve the oxidative stability of canola oil at three concentrations (100, 200, and 300 ppm). The results of peroxide value and anisidine index tests (as initial and secondary oxidation indicators, respectively) showed that the nano‐encapsulation improved the antioxidant effect of *M. aquatica* when compared with free extract in canola oil. In a comparative approach, the best sample was obtained from the LPG‐assisted nano‐encapsulated extract (200 ppm) due to the release of more phenolic compounds. The findings from this study showcase how nano‐encapsulation enhances the efficacy of antioxidants in edible oils.

## Graphical abstract

Thus, the LPG‐assisted nano‐encapsulated phenolic extract of *Mentha aquatica* was used to improve the oxidative stability of canola oil at three concentrations (100, 200, and 300 ppm). Peroxide value (PV) and anisidine value (AV) assessments showed that nano‐encapsulation improved *M. aquatica* extract's antioxidative power. In a comparative approach, the best sample resulted from the LPG‐assisted nano‐encapsulated extract (200 ppm) due to the release of more phenolic compounds.

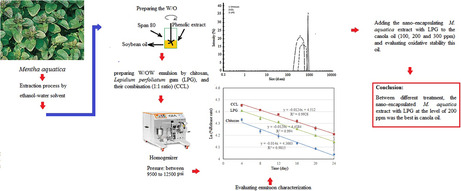

## 1 INTRODUCTION

Plants of the *Lamiaceae* family exist in different parts of Iran. *Mentha* is an important genus of this family and comprises many medicinal plants and suitable sources of essential oils (Wani et al., 2022). *Mentha aquatica*, known as Oyji in Iranian folklore medicine, is a species of this genus native to the northern regions of Iran and usually inhabits the banks of shallow water streams. The leaves of this plant are used as an edible vegetable and food flavoring agent in the north of the country. Consumption of the *Mentha* family has attracted scientific attention due to their rich phenolic profile. The mint family is known for its antioxidant, antimicrobial, and medicinal properties, mainly because of essential oils and phenolic acids (Kamkar et al., 2010; Roshanpour et al., 2021).

The oxidation of oils and fats is one of the greatest challenges that can lead to food spoilage and generate unpleasant smells, tastes, and inappropriate colors while reducing the nutritional value and undermining consumption safety (Azizi et al., 2021; Chailangka et al., 2023; Lee et al., 2021; Ugarte‐Espinoza et al., 2021). Oxidation products, such as hydroperoxides, hydroxyl radicals, and monovalent oxygen are harmful to human health and cause damage to biological cells (Amiri et al., 2021; Choudhary et al., 2023; Gonzalles et al., 2021). In turn, the damage to biological cells causes cardiovascular and neurological complications and diseases related to premature aging (Iqbal & Bhanger, 2007; Mohdaly et al., 2010). Antioxidants usually provide preventive measures against the oxidation of lipids and protect against damages caused by free radicals. In the food industry, synthetic and natural antioxidants are commonly used to enhance the oxidation stability of different food products, such as edible oils (Erkan et al., 2008; Horrillo et al., 2022; Mahdavi & Ariaii, 2021). Research has proven that synthetic antioxidants have toxic and carcinogenic effects, and thus, many developed countries have opted to restrict the use of these antioxidants (Belmonte et al., 2021; González‐Montelongo et al., 2010).

Plant extracts rich in natural antioxidants can be incorporated into different food products as an alternative to synthetic antioxidants (Mohdaly et al., 2010). Plant‐derived extracts are important sources of natural antioxidant components, such as polyphenols (Generalić Mekinić et al., 2014; Kozłowska & Gruczyńska, 2018; Nieva‐Echevarría et al., 2015; Oliveira et al., 2018). However, various problems have restricted the incorporation of natural antioxidants into food products such as low solubility, low accessibility, low penetration, and rapid destruction by environmental factors (Mohamadi et al., 2023; Tang et al., 2021). In this regard, microencapsulated and nano‐encapsulated forms of phenolic compounds are points of interest to overcome these limitations (Fang & Bhandari, 2010; Khazaei et al., 2014; Munin & Edwards‐Lévy, 2011).

Nano‐encapsulated plant extracts can be added to other food ingredients in formulations, making it easier to produce, use, store, or transfer them into food (Fang & Bhandari, 2010; Munin & Edwards‐Lévy, 2011; Roshanpour et al., 2021). Recently, protein and polysaccharide materials have been used to nano‐encapsulate natural compounds, such as antimicrobials and antioxidants, thereby increasing the functional characteristics of effective compounds and improving their controlled release (Jamshidi et al., 2020). As a natural polysaccharide, chitosan has many uses, including as a coating in micro‐ and nano‐encapsulation in the food and pharmaceutical industries (Jamshidi et al., 2020; Sahoo et al., 2009). Also, the use of gums in the nano‐encapsulation of natural compounds is gradually expanding. *Lepidium perfoliatum* seed gum, a versatile gum in Iran, has found utility in diverse research endeavors and has been employed for nano‐encapsulation purposes (Dehghan et al., 2020; Jamshidi et al., 2020). *Lepidium perfoliatum* seed is egg‐shaped and brown with a coating of co‐mucilage. This seed produces mucilage when soaked in water, which has a relatively high viscosity and can stabilize dispersed phase droplets (Dehghan et al., 2020; Koocheki et al., 2009). Roshanpour et al. (2021) investigated the phenolic extract of *Mentha piperita* using chitosan and *Alyssum homolocarpum* gum nano‐encapsulation. Their results showed that using these two combinations together (1:1) improved the antioxidant effect of the extract in soybean oil.

Therefore, considering the mentioned cases and the lack of any research regarding the nano‐encapsulation of *M. aquatica* extract, this research aimed to investigate the effect of the nano‐encapsulation process on the antioxidant effect of the phenolic extract obtained from *M. aquatic*. For this purpose, nano‐emulsions were created with *L. perfoliatum* gum (LPG) and chitosan. Ultimately, the efficiency of the nano‐encapsulated extracts was examined on the oxidative stability of canola oil and compared with the efficiency of tert‐butylhydroquinone (TBHQ). Also, pure canola oil without added antioxidants was used as a control.

## 2 MATERIALS AND METHODS

### 2.1 Materials

The *M. aquatica* was prepared by Vasteriosh company in Sari City (Mazandaran, Iran). First, the samples were cleaned manually, then dried in the shade, ground, and turned into powder. The resulting powder was subjected to the extraction process to prepare the phenolic extract. In order to prepare *L. perfoliatum* gum, 10 kg of seeds were purchased from Tabib Daru Company in Shiraz. With the cooperation of Ghoncheh Sari Oil Compan, refined canola oil without antioxidants was prepared for this research. Merck and Sigma–Aldrich companies obtained the required solvents and chemicals.

### 2.2 Extraction process

In order to prepare the phenolic extract, 100 g of powder of *M. aquatica* was mixed in 500 mL ethanol/water solvent (1:1). Then the samples were placed in an ultrasonic bath (35 kHz, for 27.5 min at 45°C) (Estakhr et al., 2020; Roshanpour et al., 2021).

### 2.3 Extraction of *L. perfoliatum* seed gums

*Lepidium perfoliatum* seed gum was extracted at the optimum conditions (seed‐to‐water ratio 1:30, pH: 8, 48°C), according to Jamshidi et al. (2020) and Koocheki et al. (2009). Briefly, *L. perfoliatum* seeds were dispersed in preheated deionized water (48°C), and the slurry of seed water was mixed continuously during the extraction period (1.5 h) while the temperature was kept constant (48°C) and pH was monitored and adjusted at 8. Finally, the seeds were discarded, and the slurry was dried in an oven (45°C), milled, and sieved by a mesh 18 sifter.

### 2.4 Preparation of biopolymer solutions

Wall materials solution (0.5% w/v) was prepared by dissolving *L. perfoliatum* seed gum in the distilled water and chitosan in the acetic acid solution (1%) and stirring for 30 min at room temperature. The solutions were kept in the refrigerator for 24 h to complete the hydration and disengage the bubbles (Jamshidi et al., 2020).

### 2.5 Preparation of nano‐emulsions

The W/O/W two‐layer nano‐emulsions were prepared using two emulsion‐forming steps. First, W/O emulsion was prepared by dropwise adding 7% *M. aquatica* extract in a continuous phase containing 25% span 80 and 68% soybean oil without antioxidants. In the second emulsification phase, the W/O initial emulsion was coated with biopolymers prepared to produce W/O/W double emulsions. As a result, 30% initial W/O emulsion was added to 70% prepared bipolymers and homogenized at 10°C for 5 min at 13,709 g and then at 30,845 g for 8 min. Then, the homogenizer (Avastin EmulsiFlex C3, ATA) was used at a pressure of 9500–11,500 psi in 3, 5, and 7 cycles (90 s each) to reduce the particle size and to stabilize the emulsion (Estakhr et al., 2020).

### 2.6 Particle size measurement and ζ‐potential

Particle distribution, average particle size (Z‐average), and polydispersity Index (PDI) were done by dynamic light scattering (DLS) instrument at 25°C (Tavakoli et al., 2021). The method used by Roshanpour et al. (2021) was used to measure the ζ‐potential.

### 2.7 Freeze drying of nano‐emulsions

The nano‐emulsions prepared from the previous step were placed in a freezer at −50°C for one night and then dried in a freeze dryer at a pressure of 0.09 bar and 0.01°C for 48 h, respectively. Ultimately, these samples were turned into powder using a mortar (Delfanian et al., 2018).

### 2.8 Encapsulation efficiency and total phenolic content

In order to measure the encapsulation efficiency, the method described by Robert et al. (2010) was used. For this purpose, 0.5 g of nano‐encapsulated powders were mixed with 2 mL of ethanol‐methanol (1:1) and vortexed for 2 min. The resulting mixture was then straightened with Watchell's No. 1 filter paper. The amount of phenolic compounds was determined spectrophotometrically using Folin‐Ciocalteau's reagent, according to the method described by Delfanian et al. (2018). A calibration curve of gallic acid was performed over the concentration range of 0.04–0.40 mg/mL. The formula for calculating the Encapsulation efficiency is as follows:

$$\text{Encapsulation efficiency}\;\left(\%\right)=100-\left(\left(\frac{P_{2}}{P_{1}}\right)\times 100\right)$$

P_2_: surface phenolic compounds; P_1_: theoretical total polyphenols content.

### 2.9 Release kinetics

The stability of the encapsulated powder was determined based on the release rate of phenolic compounds present in the inner part of the W/O/W nano‐emulsion. Approximately 12 g of nano‐sized samples were poured into dark glass containers. They were then placed in an oven at 30°C for 24 days. At the end of each 4 day, the amount of phenolic compounds was determined according to the method described by Estakhr et al. (2020).

The rate constant (*k*) and half‐life period (*t*_1/2_) of encapsulated powders were determined from the slope of the semi‐logarithm plotted of their remaining contents in nano‐capsules versus storage time. The half‐life of polyphenols “*t*_1/2_”, which is defined as the time of a reduction of 50% of their initial values in the capsules, was calculated from the slope of the curve and based on the *t*_1/2_ = 0.693/*k* (Najafi et al., 2011).

### 2.10 p‐Anisidine value and peroxide value

The peroxide value was measured according to the spectrophotometric method described by Tavakoli et al. (2019). Results were expressed in milliequivalents of peroxide per kilogram of oil. The anisidine value (AnV) was determined by measurement of the aldehydes content (ISO 6885:^2016^).

### 2.11 Statistical analysis

The experiments of this research were done in three repetitions, and the results were analyzed with the help of analysis of variance. Moreover, Excel and Slide Write software were used to design graphs and regression. Meanwhile, Duncan's test was applied to compare the mean values.

## 3 RESULTS AND DISCUSSION

### 3.1 Evaluating the nano‐emulsions

By the DLS method, the results showed droplet size distribution and polydispersity index (PDI) of the different emulsions (based on chitosan, LPG, and complex of chitosan and LPG (1:1 ratio) (CCL)). To reduce the droplet size and homogenization, a pressure of 11,000 psi was selected for smaller droplet sizes (Table 1). At pressures higher than 11,000 psi, the re‐coagulation of droplets was observed, which made their droplet size larger. To homogenize the emulsions, this pressure was applied for 3, 5, and 7 cycles (each cycle lasting for 90 s). Previous research confirmed that if the input pressure of the homogenizer becomes higher than optimal, the emulsion droplet size will increase (Mohammadi et al., 2015; Taghvaei et al., 2014). The smallest z‐average size of the emulsion droplets was created in the 5‐cycle treatment, which was 68.2, 65.3, and 62.2 nm in the chitosan, CCL, and LPG groups, respectively (Table 2). The results showed that using LPG had an optimal effect on the droplet size of emulsions, followed by CCL and chitosan. An investigation by Delfanian et al. (2018) found that using Hi‐Cap 100 alone resulted in the best z‐average size. However, when combined with other materials, the outcome was negatively affected. The emulsifying properties of coating materials, due to several variables, such as plasticity, surface activity, and surface absorption rate on the droplet surface, can cause differences in the droplet size of emulsions (Hosseinialhashemi et al., 2021; Jamshidi et al., 2020).

**TABLE 1:** The particle size of W/O/W double emulsions stabilized by different wall materials at three‐time cycles and five pressures (psi).

Sample	Pressure	Time cycle		
		3	5	7
Chitosan	9500	200 ± 1.7^a^	180.3 ± 1.6^b^	199.9 ± 1.2^a^
	10,000	169.1 ± 1.3^a^	152.4 ± 1.5^c^	162.7 ± 1.7^b^
	10,500	137.8 ± 0.2^b^	105.6 ± 1.9^c^	142.2 ± 1.3^a^
	11,000	113.5 ± 0.2^a^	68.2 ± 0.3^c^	97.0 ± 0.2^b^
	11,500	197.3 ± 0.5^a^	173.2 ± 1.4^c^	183.9 ± 0.8^b^
CCL	9500	195.8 ± 1.2^a^	152.5 ± 1.5^c^	162.1 ± 0.54^b^
	10,000	162.7 ± 0.7^a^	110.1 ± 1.2^c^	132.4 ± 0.4^b^
	10,500	127.3 ± 0.6^a^	88.3 ± 1.1^c^	110.1 ± 0.2^b^
	11,000	110.2 ± 0.3^a^	65.3 ± 0.4^c^	93.4 ± 0.3^b^
	11,500	120.4 ± 0.2^b^	97.3 ± 1.3^c^	122.2 ± 0.3^a^
LPG	9500	205.5 ± 0.5^a^	157.1 ± 1.1^c^	200.6 ± 0.4^b^
	10,000	187.7 ± 0.4^a^	133.2 ± 1.3^c^	154.3 ± 0.6^b^
	10,500	127.1 ± 0.2^a^	97.6 ± 1.4^c^	103.5 ± 0.5^b^
	11,000	104.4 ± 0.2^a^	62.2 ± 0.3^c^	88.1 ± 0.5^b^
	11,500	123.1 ± 0.8^a^	96.6 ± 1.1^c^	115.6 ± 1.4^b^

*Note*: Means ± SD (standard deviation) within a row with the same lowercase letters is not significantly different at *p* < .05.

Abbreviations: CCL, complex of chitosan and LPG (1:1); LPG, *Lepidium perfoliatum* gum.

**TABLE 2:** Particle size, polydispersity index (PDI), and zeta potential of W/O/W double emulsions stabilized by different wall materials at three‐time cycles.

Sample	Particle size (nm)			PDI			ζ‐potential (mV)
	3	5	7	3	5	7	
Chitosan	113.5 ± 0.2^a^	68.2 ± 0.3^a^	97.0 ± 0.2^a^	0.47 ± 0.003^b^	0.41 ± 0.004^b^	0.4 ± 0.004^c^	26.3 ± 0.2^a^
LPG	104.4 ± 0.2^c^	62.2 ± 0.3^c^	88.1 ± 0.5^c^	0.46 ± 0.003^c^	0.36 ± 0.004^a^	0.42 ± 0.002^b^	−35.3 ± 0.2^c^
CCL	110.2 ± 0.3^b^	65.3 ± 0.4^b^	93.4 ± 0.3^b^	0.55 ± 0.004^a^	0.36 ± 0.005^a^	0.48 ± 0.003^a^	18.1 ± 0.3^b^

*Note*: Means ± SD (standard deviation) within a column with the same lowercase letters is not significantly different at *p* < .05.

Abbreviations: CCL, complex of chitosan and LPG (1:1); LPG, *Lepidium perfoliatum* gum.

Dehghan et al. (2020) used nano‐encapsulated orange peel essential oil with *Lepidium sativum* and *Salvia macrosiphon* gums and evaluated their effects on the properties of soybean oil. The results showed that the combined use of these two gums (1:1) caused the greatest reduction in the droplet size of emulsions. Also, Jamshidi et al. (2020) used two coatings of chitosan and *L. perfoliatum* gum, nano‐encapsulated unsaponifiable matters of bran oil from two different rice varieties (Tarom and Fajr). After evaluating their different characteristics, droplet sizes of nano‐encapsulated emulsions with chitosan were reportedly 199 and 233 nm, respectively, and with *L. perfoliatum*, 146 and 216 nm, respectively. In the current research, however, the droplet size of emulsions was smaller, although with similar coatings. These differences can be explained by the effect of nano‐encapsulated materials, that is, the differences between unsaponifiable matters of the rice bran oil varieties and *M. aquatica* extract.

**FIGURE 1:**
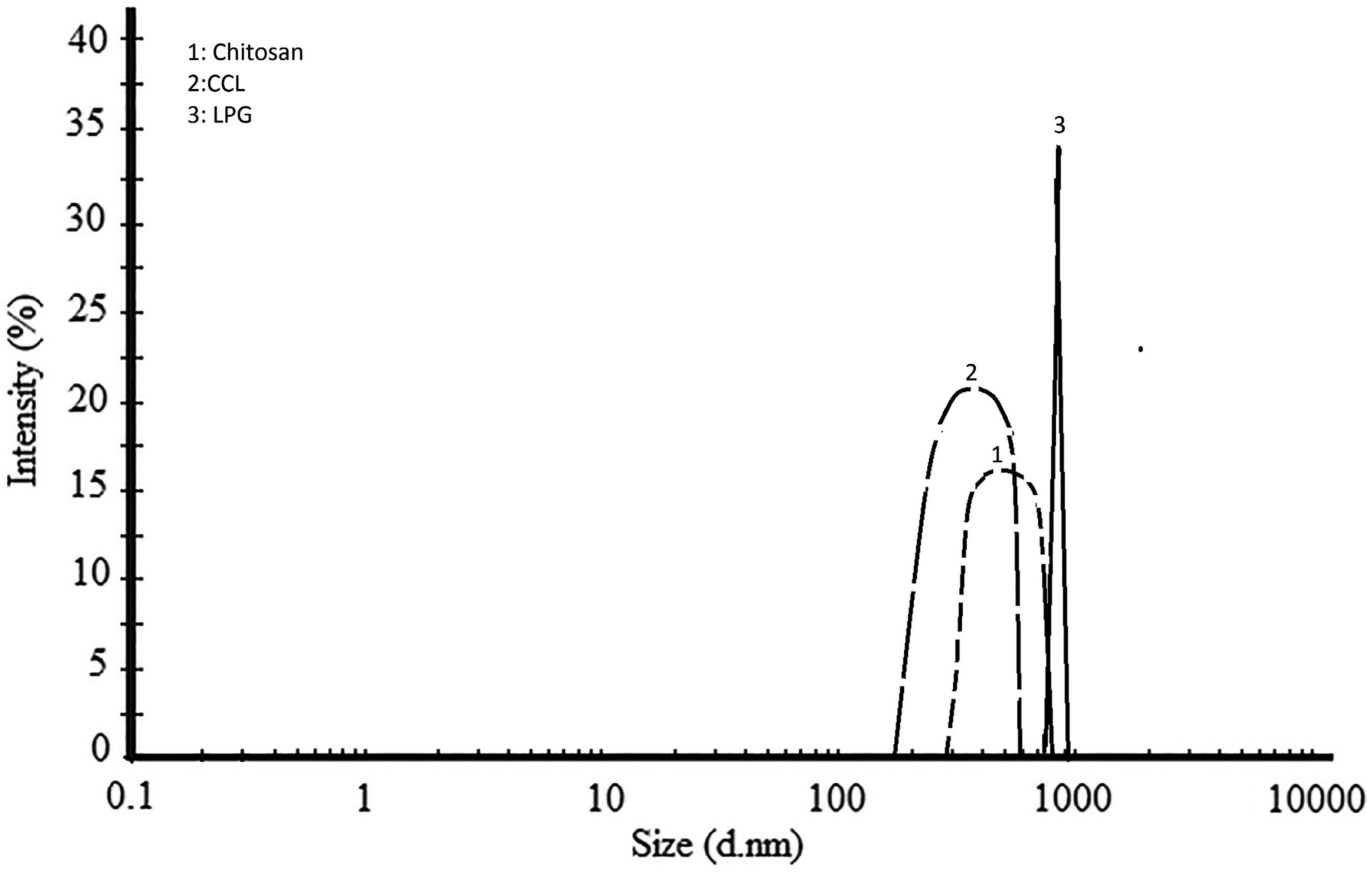
The particle size distribution of W/O/W emulsions of single‐layer chitosan, LPG, and two‐layer CCL. CCL, complex of chitosan and LPG (1:1); LPG, *Lepidium perfoliatum* gum.

The z‐average size parameter is a physical measure. Besides this factor, the intensity distribution is used to have greater accuracy (Delfanian et al., 2018). In Figure 1, the intensity distribution parameter is used to depict the droplet size distribution curve of the emulsion samples. An analysis of the curve revealed that the emulsions prepared from Chitosan, CCL, and LPG had peaks at 500, 380, and 830 nm, respectively. This indicates that the droplet size distribution was consistent across all samples. Also, similar to particle size, these values were observed in the fifth cycle. In the intensity distribution curve, the lower bandwidth indicated a lower range of changes in the diameter of the emulsion particles. Thus, the sample created with LPG coating was the most uniform and suitable emulsion in the present research, followed by samples created with CCL and chitosan. In previous research cases where different gums were used, a combination of two different coatings performed best. Estakhr et al. (2020) and Roshanpour et al. (2021) reported that the combination of chitosan with locust bean and Qodume Shirazi gums (1:1) generated the most uniform nano‐emulsions. Also, Tavakoli et al. (2021) created optimal nano‐emulsions from a combination of *L. sativum* and *S. macrosiphon* seeds (1:1). However, several emulsions were made with a single‐layered coating and had a smaller average droplet size than the combined double‐layered coating (Delfanian et al., 2018; Mohammadi et al., 2016).

Table 2 shows the different emulsions' polydispersity index (PDI) from 0 to 1. It indicates the uniformity of the dispersions. When the PDI value is close to 0, it indicates the dispersion particle size homogeneity, whereas PDI values greater than 0.5 indicate non‐uniform conditions (Delfanian et al., 2018; Lutz et al., 2009). According to Table 2, the PDI of nano‐emulsions was less than 0.5, indicating the uniformity of size distribution and, thus, favorability of the nano‐particle production process. Only in the CCL‐assisted sample, the value of this index was 0.55 in the third cycle. According to this evaluation, the nano‐emulsions created with LPG were the most uniform compared to the other treatment groups, followed by chitosan and CCL nano‐emulsions, respectively. A related study reported that the amount of PDIfor nano‐emulsions produced using locust bean gum and a combination of locust bean gum with chitosan in the third to seventh cycles ranged between 0.378–0.439 and 0.256–0.344, respectively (Estakhr et al., 2020), which were less than 0.5. Tavakoli et al. (2021) reported that the amount of PDI of nano‐emulsions coated with *L. sativum* gum and *S. macrosiphon* gum, as well as a mixture of these two gums (1:1), ranged from 0.0996 to 0.1198, from 0.1002 to 0.1112, and from 0.072 to 0.091, respectively (Tavakoli et al., 2021). It was reported that the combination of the two gums was most optimal. In another study, Delfanian et al. (2018) described an emulsion created with soy protein isolate‐basil seed gum with PDI values of less than 0.3.

Similar to the present research, chitosan, and *L. perfoliatum* were used as coatings for nano‐encapsulation, showing that the PDI values of unsaponifiable matters of rice bran oil (Tarom and Fajr) with *L. perfoliatum* nano‐encapsulation were 0.36 and 0.31, respectively. In contrast, the PDI values were 0.44 and 0.32, respectively, when using chitosan (Jamshidi et al., 2020). With the same nano‐encapsulation coatings, the results differed when changing the variety of rice. Also, Dehghan et al. (2020) used nano‐encapsulated orange peel essential oil using *L. perfoliatum* and reported a PDI value of 0.662, which was higher than that of the present study.

The ζ‐potential is an important index for the evaluation of colloidal (Jones et al., 2010). Among the two‐layered nano‐emulsions of droplets made of the different coatings (Table 2), it is observed that the ζ‐potential in emulsion droplets covered with LPG, chitosan, and CCL were −35.3, 26.3, and 18.1 mV, respectively. Since a higher ζ‐potential means that the emulsion is stable, the LPG was best in this regard, followed by chitosan and CCL.

An investigation reported that the ζ‐potential of locust bean gum, chitosan, and their combination (1:1) was −41.05, 25.97, and −9.2, respectively (Estakhr et al., 2020). Roshanpour et al. (2021) also determined the ζ‐potential in *M. piperita* extract, nano‐encapsulated with *A. homolocarpum* gum, chitosan, and their combination (1:1), reporting values of −37, 29, and 20 mV, respectively. Tavakoli et al. (2021) reported that the ζ‐potential of *L. sativum* and *S. macrosiphon* was −18.4 and −18, respectively.

Nano‐encapsulation usually improves antioxidant effect (Razali et al., 2012). The encapsulation efficiency of each treatment group is shown according to the amount of total polyphenols that remained during the 24 days at 30°C (Table 3). The type of coating influences the encapsulation efficiency. The highest initial efficiency of nano‐encapsulation was observed in phenolic extracts coated with LPG (85.9%), followed by CCL (82.1%) and chitosan (76.1%). Jamshidi et al. (2020) described the encapsulation efficiency of unsaponifiable bran oil from two rice varieties (Tarom and Fajr) using chitosan and LPG. The encapsulation efficiency ranged from 74.36% to 80.35% and from 78.18% to 83.09%, respectively, in the bran oils of Tarom and Fajr rice varieties, which were almost similar to the results of the present study. Also, in another research, orange peel essential oil coated with LPG encapsulation efficiency was 85.2% (Dehghan et al., 2020). Hosseinialhashemi et al. (2021) used two gums (*Lallemantia royleana* and *Trigonella foenum‐graecum*) for the nano‐encapsulation of *P. khinjuk* phenolic extract, reporting that the encapsulation efficiency ranged from 59.1% to 63.5%. In contrast to the current study, the research conducted by Hosseinialhashemi et al. (2021) identified the combined coating of two gums as the optimal treatment. The disparity in outcomes between these two studies can be attributed to using distinct compounds in the nano‐encapsulation process, notably chitosan, which possesses distinct properties compared to gums.

**TABLE 3:** Encapsulation efficiency, regression analysis, and half‐life values of encapsulated powders produced with different wall materials during 24 days of storage at 30°C.

Sample	Storage time (day)						Parameters		
	4	8	12	16	20	24	*k* (day^−1^)	*t*_1/2_ (day)	*R* ^2^
Chitosan	76.1 ± 0.9^c^	69 ± 1^c^	66.3 ± 1.1^c^	62.4 ± 0.6^c^	59.6 ± 0.5^c^	56.8 ± 1.9^c^	0.0140	49.5	.993
CCL	82.1 ± 0.3^b^	77 ± 0.9^b^	74.8 ± 0.3^b^	70.5 ± 0.5^b^	66.5 ± 0.5^b^	63.1 ± 0.7^b^	0.0129	53.7	.992
LPG	85.9 ± 0.9^a^	82.5 ± 0.5^a^	79.5 ± 0.5^a^	75 ± 1^a^	70.8 ± 1.1^a^	67.3 ± 1.5^a^	0.0124	55.9	.993

*Note*: Means ± SD (standard deviation) within a column with the same lowercase letters are not significantly different at *p* < .05.

Abbreviations: CCL, complex of chitosan and LPG (1:1); LPG, *Lepidium perfoliatum* gum.

In the current research, the smallest decrease in encapsulation efficiency was observed in the LPG‐assisted encapsulation (21.7%), followed by the CCL‐assisted (23.1%) and chitosan‐assisted encapsulation (25.3%) (Table 3). These values are consistent with the droplet size of the nano‐emulsions. Salvia‐Trujillo et al. (2016) reported that the smaller the droplet size of emulsions, the higher the encapsulation efficiency. Tavakoli et al. (2021) reported that the encapsulation efficiency of potato extract, nano‐encapsulated with *L. sativum* and *S. macrosiphon*, decreased by 33%–45% during 40 days of storage. The best treatment was the equal combination of these two gums (1:1) for nano‐encapsulation. In the present research, the size of emulsions correlated with the encapsulation efficiency. Roshanpour et al. (2021) reported that the encapsulation efficiency of *M. piperita* extract, nano‐encapsulated with chitosan and *A. homolocarpum* gum, decreased by 19.9%–24.3%. The lowest decrease was observed when an equal combination of the two materials (1:1) was used in the coating. Estakhr et al. (2020) showed an initial encapsulation efficiency of 89% for *Ferula persica* extract, nano‐encapsulated with locust bean gum, which decreased by 19.9% during 24 days of storage at 30°C.

**FIGURE 2:**
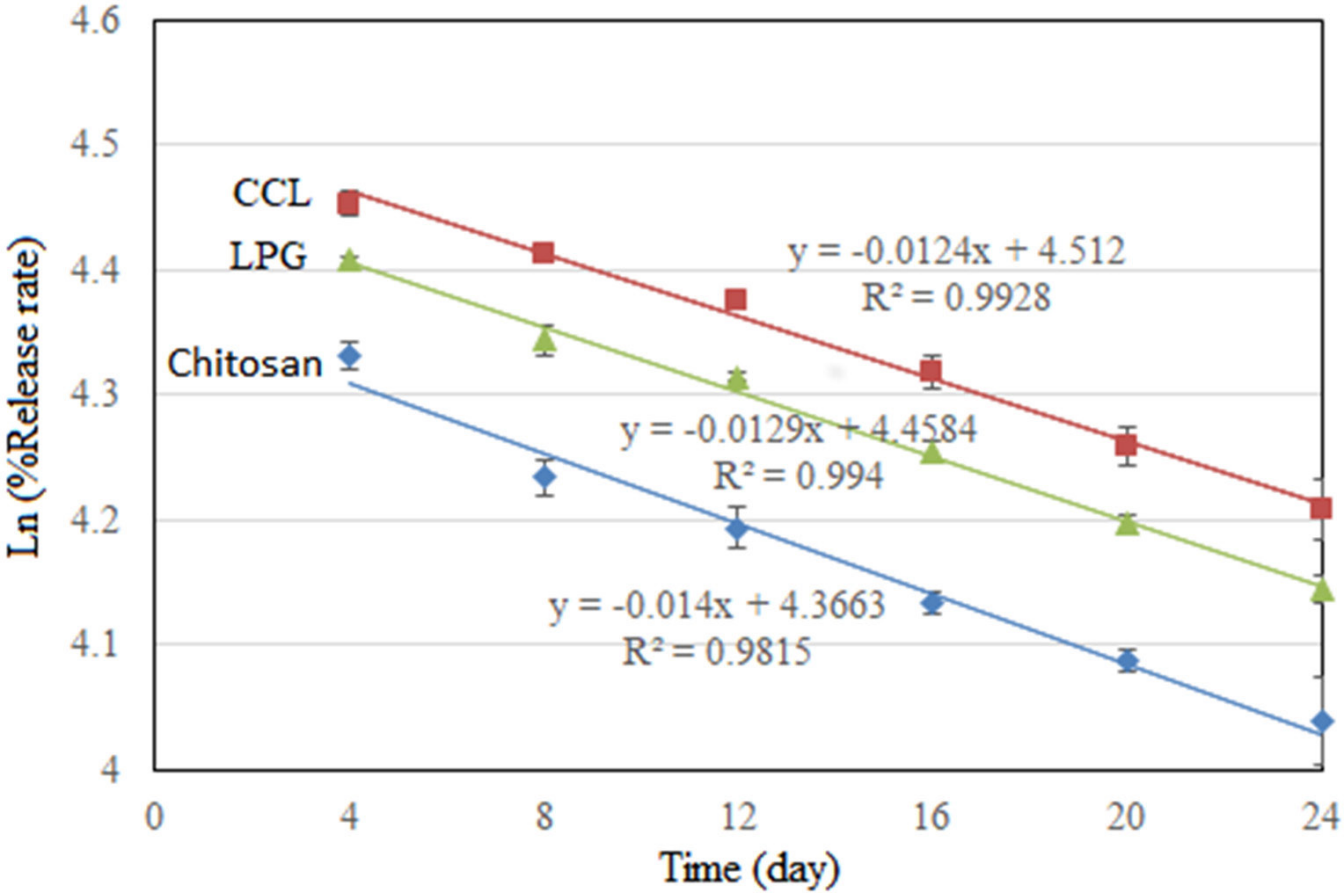
Release rate and regression equations of phenolic compounds of nanocapsules coated with LPG, chitosan, and CCL. CCL, complex of chitosan and LPG (1:1); LPG, *Lepidium perfoliatum* gum.

Table 3 and Figure 2 present the encapsulated powders' half‐life period (*t*_1/2_) and rate constant (*k*). In the case of samples subjected to chitosan, CCL, and LPG nano‐encapsulation, the rate constants were recorded as 0.014, 0.0129, and 0.0124, respectively. Correspondingly, the half‐life periods for these samples were 49.5, 53.7, and 55.9 days, respectively. It is noteworthy that the rate constant exhibited an inverse correlation with the half‐life period.

In another study involving the nano‐encapsulation of phenolic extract from *Mentha* piperita using chitosan and *A. homolocarpum* gum, the reported rate constants ranged from 0.011 to 0.0143, while the corresponding half‐life periods fell between 48.5 and 63 days (Roshanpour et al., 2021).

### 3.2 Effect of nano‐encapsulated extracts on the oxidative stability of canola oil

Its effect on the oxidative stability of edible oils should be evaluated to identify and introduce an antioxidant with appropriate potency. Thus, oil is usually subjected to intensified oxidative conditions and various experiments, such as the peroxide and p‐anisidine values over time (Zhang et al., 2010). Previous tests on the evaluation of nano‐emulsions in the present research determined that nano‐encapsulating the *M. aquatica* extract with LPG resulted in the best outcome. Therefore, among the different coatings, the effect of nano‐encapsulation extract with LPG was evaluated regarding the oxidative stability of the canola oil (24 days at 60°C). The free phenolic extract of *M. aquatica* and TBHQ were observed comparatively.

Hydroperoxides are the primary oxidation product of edible fats and oils. Due to heat, these components are converted to secondary compounds, including carbonyls (Tavakoli et al., 2019; Zhang et al., 2010). Changes in the peroxide value of various canola oil treatments are shown in Table 4. The rate of increase in the peroxide value of pure canola oil and canola oil containing 100, 200, and 300 ppm of free *M. aquatica* extract, as well as 100, 200, and 300 ppm nano‐encapsulated *M. aquatica* extract with LPG and 100 ppm TBHQ were determined as 374%, 231%, 192%, 137%, 132%, 88%, 128%, and 278%, respectively. The results showed that increasing the free *M. aquatica* extract in canola oil caused an increase in oil resistance to primary oxidation. Also, the nano‐encapsulation process positively affected the extracts' antioxidant effect, and the best sample was the nano‐encapsulated extract (200 ppm). However, unlike free extracts, increasing the amount of nano‐encapsulated extract up to 200 ppm alleviated primary oxidation and caused a decrease in hydroperoxide formation. However, increasing the concentration of the nano‐encapsulated extract from 200 to 300 ppm decreased the resistance to primary oxidation, and hydroperoxide formation increased. In the 300 ppm nano‐encapsulated extract, phenolic compounds are not released efficiently from the LPG coating. In research by Estakhr et al. (2020), increasing the concentration of nano‐encapsulated extract with locust bean gum from 100 to 300 ppm caused a decrease in hydroperoxide formation in soybean oil, whereas using chitosan coating at 200 ppm resulted in a better outcome than the concentrations of 100 and 300 ppm, thereby confirming the results of the present research. Also, the superiority of free and nano‐encapsulated extracts over TBHQ was one of the interesting results of this research. TBHQ is a very powerful antioxidant, especially at very high temperatures. At low temperatures, the oxidation mechanism differed from very high temperatures (above 150°C). Probably, at low temperatures, similar to the present research, the antioxidant effect of different *M. aquatica* extracts was superior to the efficiency of TBHQ. In other studies, similar results were reported on the activity of plant extracts and TBHQ affecting the peroxide value (Estakhr et al., 2020; Tavakoli et al., 2021).

**TABLE 4:** Effect of adding *M. aquatica* extract and nano‐encapsulated *M. aquatica* extract produced by LPG and TBHQ (100 ppm) on peroxide value of canola oil under accelerated storage at 60°C for 24 days.

Time (day)	Control	Free *M. aquatica* extract		
		100 ppm	200 ppm	300 ppm
0	2.5 ± 0.1^b^	2.7 ± 0.1^a^	2.7 ± 0.1^a^	2.7 ± 0.1^a^
4	5.0 ± 0.1^a^	4.4 ± 0.1^c^	4.1 ± 0.1^d^	3.8 ± 0.1^e^
8	5.7 ± 0.2^a^	5.0 ± 0.1^c^	4.6 ± 0.1^d^	4.2 ± 0.1^e^
12	7.6 ± 0.4^a^	5.9 ± 0.3^c^	5.4 ± 0.2^d^	4.9 ± 0.2^e^
16	8.8 ± 0.3^a^	7.0 ± 0.2^c^	6.3 ± 0.1^d^	5.4 ± 0.1^e^
20	9.6 ± 0.3^a^	7.5 ± 0.2^c^	6.8 ± 0.2^d^	5.8 ± 0.1^e^
24	11.9 ± 0.1^a^	8.9 ± 0.1^c^	7.9 ± 0.1^d^	6.4 ± 0.1^e^

Time (day)	TBHQ	Nano‐encapsulated *M. aquatica* extract produced by LPG		
		100 ppm	200 ppm	300 ppm
0	2.7 ± 0.1^a^	2.8 ± 0.1^a^	2.8 ± 0.1^a^	2.8 ± 0.1^a^
4	4.8 ± 0.1^b^	3.6 ± 0.1^f^	3.4 ± 0.1^f^	3.8 ± 0.1^e^
8	5.4 ± 0.2^b^	4.1 ± 0.1^e^	3.7 ± 0.1^f^	4.2 ± 0.1^e^
12	7.1 ± 0.4^b^	5.0 ± 0.2^e^	4.5 ± 0.2^f^	4.9 ± 0.2^e^
16	8.0 ± 0.2^b^	5.2 ± 0.1^f^	4.5 ± 0.1^h^	5.4 ± 0.1^e^
20	8.7 ± 0.2^b^	5.7 ± 0.1^e^	5.0 ± 0.1^f^	5.8 ± 0.1^e^
24	10.6 ± 0.1^b^	6.5 ± 0.1^e^	5.3 ± 0.1^f^	6.4 ± 0.1^e^

*Note*: Means ± SD (standard deviation) within a row with the same lowercase letters are not significantly different at *p* < .05.

Abbreviation: LPG, *Lepidium perfoliatum* gum.

The p‐anisidine test indicates the secondary oxidation stage and carbonyl compounds' production in edible oils (Tavakoli et al., 2019, 2021; Zhang et al., 2010). Table 5 shows the p‐anisidine value of different oil treatments during the 24 days of storage at 60°C. After heated storage, the p‐anisidine value in pure canola oil and canola oil containing 100, 200, and 300 ppm *M. aquatica* free extract, as well as 100, 200, and 300 ppm nano‐encapsulated extract with LPG and 100 ppm TBHQ, increased by 71%, 48%, 41%, 35%, 37%, 29%, 32% and 48%, respectively. Similar to the peroxide value, an increase in the concentration of free extracts improved the stability of canola oil against secondary oxidation. The nano‐encapsulation of the extract also inhibits the formation of secondary oxidation products in canola oil.

**TABLE 5:** Effect of adding *M. aquatica* extract and nano‐encapsulated *M. aquatica* extract produced by LPG and TBHQ (100 ppm) p‐anisidine value of canola oil under accelerated storage at 60°C for 24 days.

Time (day)	Control	Free *M. aquatica* extract		
		100 ppm	200 ppm	300 ppm
0	1.3 ± 0.1^a^	1.3 ± 0.1^a^	1.3 ± 0.2^a^	1.2 ± 0.1^a^
4	1.5 ± 0.05^a^	1.3 ± 0.04^c^	1.2 ± 0.04^d^	1.1 ± 0.03^e^
8	1.6 ± 0.04^a^	1.4 ± 0.03^c^	1.3 ± 0.05^d^	1.2 ± 0.03^e^
12	1.8 ± 0.03^a^	1.4 ± 0.02^c^	1.3 ± 0.04^d^	1.2 ± .03^e^
16	1.9 ± 0.04^a^	1.6 ± .03^c^	1.5 ± .02^d^	1.3 ± .02^e^
20	2.0 ± 0.04^a^	1.7 ± 0.03^c^	1.6 ± 0.04^d^	1.5 ± 0.03^e^
24	2.2 ± 0.03^a^	1.9 ± 0.02^c^	1.8 ± 0.03^d^	1.7 ± 0.02^e^

Time (day)	TBHQ	Nano‐encapsulated *M. aquatica* extract produced by LPG		
		100 ppm	200 ppm	300 ppm
0	1.4 ± 0.1^a^	1.2 ± 0.1^a^	1.14 ± 0.2^a^	1.06 ± 0.2^a^
4	1.4 ± 0.04^b^	1.1 ± 0.05^e^	1.0 ± 0.02^f^	0.9 ± 0.05^g^
8	1.5 ± 0.02^b^	1.1 ± 0.04^f^	1.1 ± 0.03^f^	1.0 ± 0.02^g^
12	1.7 ± 0.03^b^	1.2 ± 0.02^f^	1.2 ± 0.02^f^	1.1 ± 0.02^g^
16	1.8 ± 0.03^b^	1.3 ± 0.04^f^	1.2 ± 0.03^g^	1.1 ± 0.03^h^
20	1.9 ± 0.02^b^	1.4 ± 0.02^f^	1.3 ± .02^g^	1.2 ± 0.03^h^
24	2.1 ± 0.03^b^	1.6 ± 0.03^f^	1.5 ± 0.03^g^	1.4 ± 0.03^h^

*Note*: Means ± SD (standard deviation) within a row with the same lowercase letters are not significantly different at *p* < .05.

Abbreviation: LPG, *Lepidium perfoliatum* gum.

**FIGURE 3:**
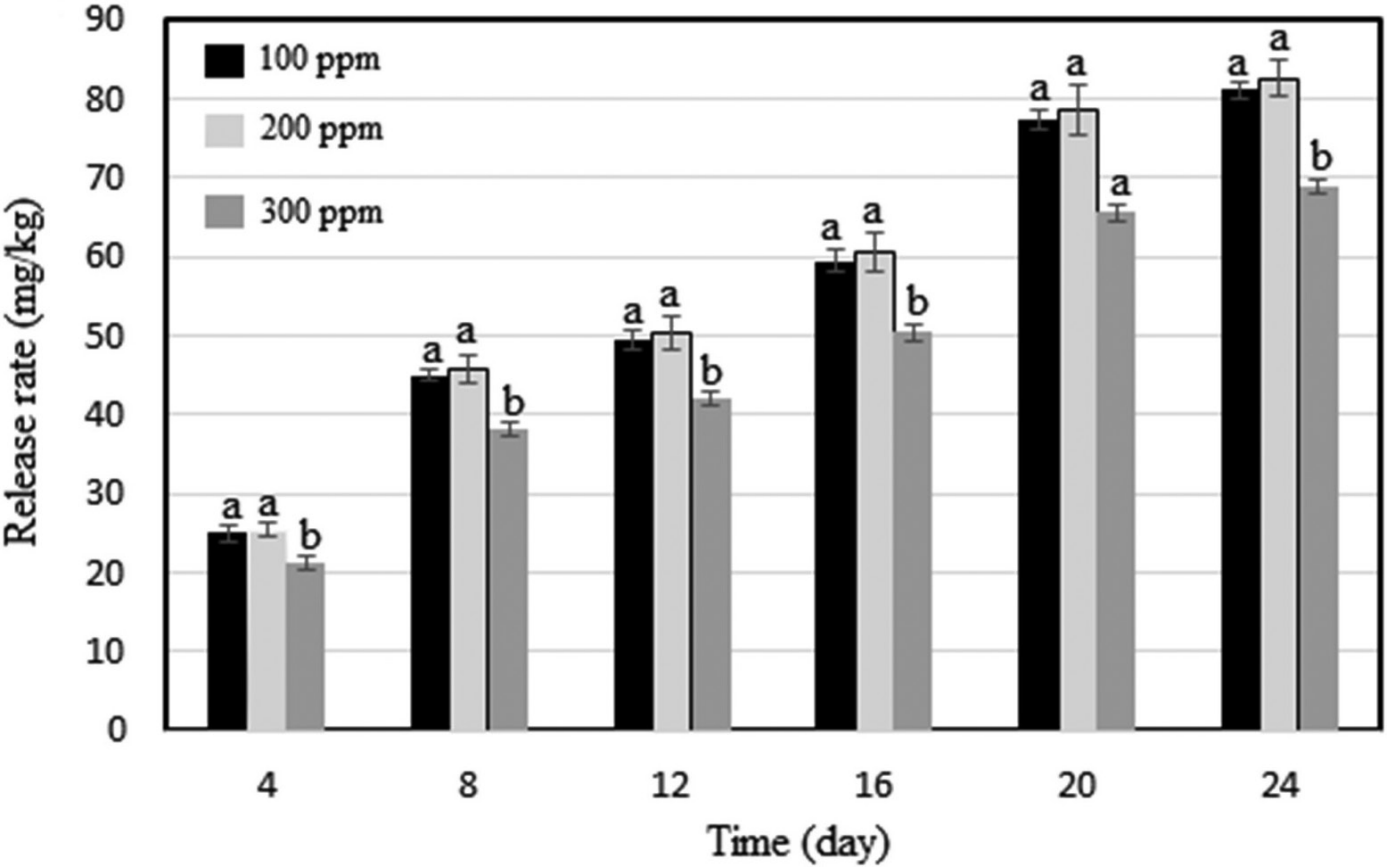
The release rate of phenolic compounds in different oil samples from encapsulated powders produced by LPG at levels of 100, 200, and 300 ppm. LPG, *Lepidium perfoliatum* gum.

Furthermore, the nano‐encapsulated extracts, akin to the outcomes of the peroxide value test, the nano‐encapsulated extract at a concentration of 200 ppm emerged as the optimal choice. Most treatments exhibited enhancements in the oxidative stability of canola oil throughout the storage duration. As depicted in Figure 3, the escalation in the phenolic compound concentration during the nano‐encapsulation process, progressing from 200 to 300 ppm, did not yield a favorable release of these compounds. This phenomenon identified the nano‐encapsulated 200 ppm extract as the most effective solution.

A comparative assessment of the peroxide and anisidine values showed that the oil samples were subjected to severe primary oxidation, whereas secondary oxidation was not as severe. The lowest and highest increase in anisidine value was 29% and 71%, respectively, whereas the lowest and highest values in peroxide value were 132% and 374%, respectively. Estakhr et al. (2020) reported that soybean oil's lowest increase in anisidine value occurred when 300 ppm *F. persica* extract was nano‐encapsulated with locust bean gum and chitosan. In other investigations, similar results were reported on the positive effects of nano‐encapsulation on the antioxidant effect of extracts (Carneiro et al., 2013; Esfanjani et al., 2015; Mohammadi et al., 2015).

Through oxidative stability tests (peroxide value and anisidine index), it was revealed that the LPG‐assisted nano‐encapsulated phenolic extract (200 ppm) was the best treatment for preventing oxidation in canola oil. Furthermore, the release of phenolic compounds from LPG‐assisted nano‐encapsulated extracts (100, 200, and 300 ppm) was evaluated during 24 days of heated storage (60°C) (Figure 3). Among the LPG‐assisted nano‐encapsulations, the phenolic compounds exhibited the highest release rate into canola oil when the nano‐encapsulated extracts contained 200 ppm. The release was comparatively slower in the case of 100 ppm nano‐encapsulated extracts, and the slowest release was observed with the 300 ppm nano‐encapsulated extracts. This was consistent with the peroxide value and anisidine index. The nano‐encapsulated extract (200 ppm) had the highest release of phenolic compounds. Thus, it could prevent the oxidation of canola oil during storage. In a similar study, Estakhr et al. (2020) reported a decrease in the release rate of phenolic compounds from nano‐encapsulated *F. persica* extract when higher extract concentrations were used, contrary to the results of the present study. Estakhr et al. (2020) further explained that extracts coated with locust bean gum and chitosan (1:1) were more efficient in oxidative stability tests, indicating an efficient release of phenolic compounds into soybean oil. However, the nano‐encapsulated extract that causes the best oxidative stability in edible oils does not always have the highest release rate of phenolic compounds. According to the available literature, nano‐encapsulation increases the stability of phenolic compounds compared to the absence of nano‐encapsulation n (Carneiro et al., 2013; Esfanjani et al., 2015; Tavakoli et al., 2021). However, other research cases showed that nano‐encapsulation negatively affects the antioxidant effect of phenolic extracts (Delfanian et al., 2018).

## 4 CONCLUSION

The process of nano‐encapsulation for the *M. aquatica* extract involved the utilization of chitosan and LPG coatings. Subsequently, the nano‐encapsulated extract underwent a thorough evaluation to assess its antioxidant efficacy. Notably, in this study, the LPG coating proved particularly effective in generating the best nano‐emulsion. Upon subjecting the nano‐encapsulated extract to oxidative stability tests, a significant enhancement in the antioxidant potential of the M. aquatica phenolic extract within canola oil was observed. Remarkably, the most substantial boost in oxidative stability occurred when the M. aquatica extract was nano‐encapsulated with LPG at 200 ppm. This favorable outcome can be attributed to the substantial release of phenolic compounds into the canola oil. In light of these findings, nano‐encapsulating antioxidant compounds stand out as a highly suitable technique for elevating their antioxidant impact within food products—particularly in preserving food quality during storage. As a forward‐looking suggestion, exploring the stability and efficacy of nano‐encapsulated antioxidant compounds under conditions involving intense thermal processes, such as frying, could be a promising avenue for future research inquiries.

## AUTHOR CONTRIBUTIONS

**Javad Tavakoli:** Investigation (equal); resources (equal); supervision (equal); validation (equal); visualization (equal); writing – original draft (equal); writing – review and editing (equal). **Habib Abbasi:** Conceptualization (equal); formal analysis (equal); funding acquisition (equal); investigation (equal); methodology (equal); writing – original draft (equal). **Sara Gashtasebi:** Data curation (equal); formal analysis (equal); investigation (equal); methodology (equal); resources (equal); validation (equal); visualization (equal); writing – original draft (equal). **Mohsen Salmanpour:** Funding acquisition (equal); investigation (equal); methodology (equal); project administration (equal); validation (equal); visualization (equal); writing – original draft (equal); writing – review and editing (equal). **Amin Mousavi Khaneghah:** Funding acquisition (equal); project administration (equal); visualization (equal); writing – original draft (equal); writing – review and editing (equal).

## CONFLICT OF INTEREST STATEMENT

The authors declare no conflicts of interest.

## ETHICS STATEMENT

None declared.

## CONSENT TO PARTICIPATE

The authors declare their consent to participate in this article.

## CONSENT TO PUBLISH

The authors declare their consent to publish this article.

## Data Availability

None declared.
